# Soft Coulomb gap and asymmetric scaling towards metal-insulator quantum criticality in multilayer MoS_2_

**DOI:** 10.1038/s41467-018-04474-4

**Published:** 2018-05-24

**Authors:** Byoung Hee Moon, Jung Jun Bae, Min-Kyu Joo, Homin Choi, Gang Hee Han, Hanjo Lim, Young Hee Lee

**Affiliations:** 1 Center for Integrated Nanostructure Physics, Institute for Basic Science (IBS), Suwon, 16419 Republic of Korea; 2 Department of Energy Science, Sungkyunkwan University, Suwon, 16419 Republic of Korea; 3 Institute for Basic Science (IBS), Daejeon, 34047 Republic of Korea

## Abstract

Quantum localization–delocalization of carriers are well described by either carrier–carrier interaction or disorder. When both effects come into play, however, a comprehensive understanding is not well established mainly due to complexity and sparse experimental data. Recently developed two-dimensional layered materials are ideal in describing such mesoscopic critical phenomena as they have both strong interactions and disorder. The transport in the insulating phase is well described by the soft Coulomb gap picture, which demonstrates the contribution of both interactions and disorder. Using this picture, we demonstrate the critical power law behavior of the localization length, supporting quantum criticality. We observe asymmetric critical exponents around the metal-insulator transition through temperature scaling analysis, which originates from poor screening in insulating regime and conversely strong screening in metallic regime due to free carriers. The effect of asymmetric scaling behavior is weakened in monolayer MoS_2_ due to a dominating disorder.

## Abstract (web summary)

The interplay between strong interactions and presence of disorder makes atomically thin transition metal dichalcogenides an ideal platform to study phase transitions and critical phenomena. Here, the authors observe asymmetric critical exponents around the metal-insulator-transition of multilayer MoS_2_.

## Introduction

The first experimental observation of possible metal-insulator transition (MIT) in Si metal-oxide-field-effect transistors (Si-MOSFETs)^1^ is not just phenomenological but conceptual leap against the widespread belief that the metallic phase is unviable in two-dimensional (2D) systems, which is suggested in the well-known non-interacting scaling theory of localization^2^. With successive experimental evidences^3, 4^, carrier–carrier interactions are reassessed as the origin of MIT in 2D systems^5, 6^ from the viewpoint of quantum phase transition (QPT). The mechanism of MIT is usually categorized into three phenomena, i.e., Mott, Anderson, and Mott–Anderson transition upon the relative importance of interactions and disorder. When strong interactions restrict the carrier hopping among localized atomic orbitals, this leads to an interaction-driven insulating state, called Mott insulator. On the other hand, when strong disorder is introduced and dominant over interactions, carriers can be localized by the quantum interference effect. This leads to a disorder-driven insulating state, called Anderson insulator. These two mechanisms are relatively well comprehended. However, if both interactions and disorder are comparable, the interplay between two effects is predicted to yield the complexity. In general, it has been known that a soft Coulomb gap appears around the Fermi energy in a strongly disordered system due to Coulomb interactions^7^, and glassy features are likely concomitant in the deep insulating phase. However, it remains inconclusive near MIT as to how their interplay would change this trend in a critical region, although there are numerous studies typically in Si-MOSFETs due to their diffusive characters^8^. A theory predicts some unusual transport properties such as an intermediate state^9^, which has been reported experimentally and characterized as a glassy state exhibiting non-Fermi liquid behavior in the metallic phase side^10, 11^. More complications^12^ likely exist depending on the systems. The electronic Griffiths phase could be one of them^13^. The screening effect across MIT could also be modified depending on the existence of such states, thus, drastically changing critical phenomena^14^. The theoretical phase boundary between metal and Mott- or Anderson-like insulator, depending on the relative strength of disorder and interactions, suggests a disorder screening by interactions^13^, implying that Mott-like transition can occur in the presence of quite strong disorder if interactions are sufficiently large. These subjects still draw considerable interests and more data for new material systems need to be accumulated to investigate all these complexities in strongly correlated disordered systems.

Recently developed 2D layered semiconductors, transition metal dichalcogenides (TMDs), are ideal platforms for such mesoscopic system because of the coexistence of strong carrier–carrier Coulomb interactions^15^ and disorder. In addition, the degree of interaction and disorder can be further modulated by the thickness of the film, surface passivation, and substrate engineering, providing diverse physical conditions. Interactions increase in general but disorder decreases in proportion to the thickness of TMDs.

Here, we report the electrical transports and scaling analysis near MIT in multilayer MoS_2_ including monolayer. We observe that the transport in insulating phase of multilayer MoS_2_ is consistent with the Coulomb gap description, and using this picture, we further demonstrate the criticality of MIT with the power law behavior of the localization length near MIT. The predicted glass state in the metallic phase side in the presence of Coulomb gap is nonexistent or exists in the narrow region of carrier density. With this feature, the observed asymmetric critical exponents in multilayer are ascribed to the different screenings across MIT. Lastly, the MIT is attributed to be interaction-driven in multilayer and disorder-driven in monolayer, which suggests the substantial screening of disorder induced by strong correlation effects in multilayer.

## Results

### Basic device properties

**Fig. 1:**
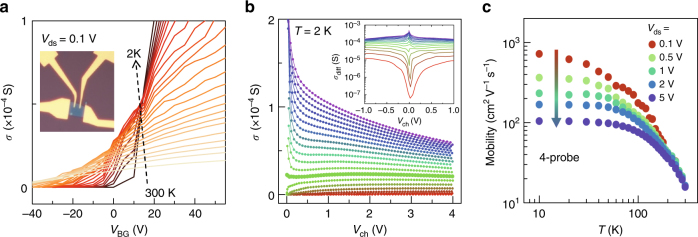
Basic transport properties and mobilities. **a** Conductivity $\sigma$ vs. backgate bias *V*_BG_ for various temperatures at a 0.1 drain-source voltage and the optical image of the device (inset). **b** $\sigma$ vs. channel voltage *V*_ch_ at 2 K for various *V*_BG_’s from −20 (bottom trace) to 70 V (top trace) in 5 V steps. Inset displays the differential conductivity ${\mathrm{d}}I_{\mathrm{D}}/{\mathrm{d}}V_{{\mathrm{ch}}}$. **c** Temperature-dependent 4-probe mobility

We fabricated a FET using a ~5-nm thick multilayer MoS_2_ with four Au electrodes, as shown in the inset of Fig. 1a. Because this thickness is much smaller than the reported coherence length, $L_{\phi}$ ~ 20 nm at temperature *T* = 10 K^16^, this system is effectively 2D. Since $L_{\phi}$decreases as *T* increases, the crossover from 2D to 3D character would appear at higher temperature. Thus, our scaling analysis will be limited below 150 K in our experiment. Figure 1a shows the backgate bias (*V*_BG_)-dependent conductivity ($\sigma$) for temperatures from 300 to 2 K. The conductivity crossing, $\Delta \sigma /\Delta T > 0$and Δ*σ*/Δ*T*<0 below ~200 K, occurs around *V*_BG_ = 10 V, which is designated as a critical field separating insulating and metallic phases. This separation can be rationalized more clearly in the field-dependent conductivity (Fig. 1b) displayed with the channel voltage $V_{{\mathrm{ch}}} = V_{{\mathrm{ds}}} - I_{\mathrm{D}}R_{\mathrm{c}}$, where $V_{{\mathrm{ds}}}$ is applied drain-source voltage, $I_{\mathrm{D}}$ drain-source current, and $R_{\mathrm{c}}$ contact resistance. Two different field dependences of $\sigma$ around *V*_BG_ = 10 V unmistakably distinguish the metallic and the insulating regimes. The inset presents the differential conductivity $\sigma _{{\mathrm{diff}}} = {\mathrm{d}}I_{\mathrm{D}}/{\mathrm{d}}V_{{\mathrm{ch}}}$ for a small range, which equally supports the determination of critical field.

At *V*_BG_ = 10 V, the critical carrier density $n_{\mathrm{c}}$ is estimated to be $\sim$3.37 × 10^12^ cm^−2^ using the simple approximation, $n_{\mathrm{c}} = C_{{\mathrm{ox}}}\left( {V_{{\mathrm{BG}}} - V_{{\mathrm{TH}}}} \right)/q$, where $C_{{\mathrm{ox}}}$ is geometrical oxide capacitance, $V_{{\mathrm{TH}}}$ threshold voltage, and *q* elementary charge, yielding *r*_s_ ~ 9.0 (~ 2.3 for Si-MOSFET for the same $n_{\mathrm{c}}$), and the Fermi temperature *T*_F_ ~ 200 K. Here, $r_{\mathrm{s}} \equiv E_{\mathrm{C}}/E_{\mathrm{F}} = m^{\ast} q^{2}g_{\mathrm{V}}/4\pi\varepsilon\varepsilon_0\hbar^{2}\sqrt {n_{2{\mathrm{D}}}}$ is the ratio of Coulomb energy *E*_C_ to kinetic energy *E*_F_ at the Fermi level, usually used as a measure of carrier–carrier interaction, where $m^{\ast}$ is effective electron mass, $g_{\mathrm{V}}$ valley degeneracy, $\varepsilon\approx 8$ relative  dielectric constant for multilayer MoS_2_^17^, *ε*_0_ vacuum dielectric constant, and $n_{2{\mathrm{D}}}$ carrier density. This value of *r*_s_(~9.0) is large for $n_{\mathrm{c}}$ ~ 3.37×10^12^ cm^−2^. This indicates that Coulomb interactions are important in this system. The actual interaction effect is expected to be larger because of the poor screening by the underlying SiO_2_ substrate ($\varepsilon _{{\mathrm{SiO}}_2} \approx 3.9$) and vacuum environment ($\varepsilon _{{\mathrm{vac}}} \approx 1$). Although the approximation may underestimate $n_{\mathrm{c}}$ value, the importance of interactions in this system is not diminished appreciably, and more importantly this does not affect the determination of critical exponents in scaling analysis as long as the linear relation between $n_{2{\mathrm{D}}}$ and $V_{{\mathrm{BG}}}$ holds (See Supplementary Note 1). One of the physical quantities that evaluate disorder is the field effect mobility $\mu _{{\mathrm{FE}}} = (1/C_{{\mathrm{ox}}})\left( {{\mathrm{d}}\sigma /{\mathrm{d}}V_{{\mathrm{BG}}}} \right)$. In this device structure, unavoidable scatterings from the substrate and the top surface strongly limit the carrier mobility. Figure 1c presents four-probe FET mobility as a function of *T* for a given *V*_ds_, where $\mu_{{\mathrm{FE}}}$ decreases as *V*_ds_ increases similar to the metallic phase (blue) in Fig. 1b. The largest $\mu_{{\mathrm{FE}}}$ at 10 K is ~720 cm^2^ V^−1^ s^−1^ at *V*_ds_ = 0.1 V (See Supplementary Note 2 for details). From these estimations, we conclude that this system is largely disordered but strongly interacting.

### Hopping conduction mechanism

**Fig. 2:**
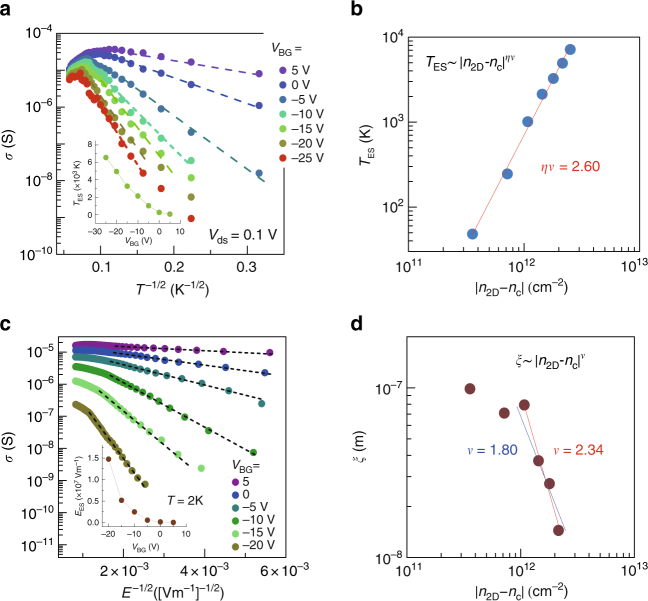
Transport properties with Coulomb gap and critical behavior. **a** $\sigma$ vs. *T*^-1/2^ and fitting parameters *T*_ES_ (inset) for selected values of *V*_BG_. **b** Logarithmic plot of *T*_ES_ as a function of carrier density difference from the critical carrier density $n_{\mathrm{c}}$, $\left| {n_{2{\mathrm{D}}} - n_{\mathrm{c}}} \right|$. **c** $\sigma$ *vs. E*^-1/*2*^, where *E* is the electric field, and fitting parameters *E*_ES_ (inset) for selected *V*_BG_ at 2 K. **d** Logarithmic plot of $\xi$ as a function of $\left| {n_{2{\mathrm{D}}} - n_{\mathrm{c}}} \right|$

The hopping conduction in the insulating phase of interacting disordered system, according to theory by Efros and Shklovskii^7^, follows as

1 $$\sigma \left( T \right) \propto {\mathrm{exp}}\left[ {\left( { - \frac{{T_{{\mathrm{ES}}}}}{T}} \right)^{1{\mathrm{/}}2}} \right],$$

where $T_{{\mathrm{ES}}} = \frac{{Cq^{2}}}{{\varepsilon \varepsilon_{0\xi} k_{\mathrm{B}}}}$. Here, $C$=6.2^18^, $\xi$ localization length, and $k_{\mathrm{B}}$ Boltzmann constant. Figure 2a shows the conductivity in a logarithmic scale as a function of $T^{-1/2}$ that is fitted with Eq. (1) for different *V*_BG_. The parameter *T*_ES_ is obtained with fairly reasonable fitting (inset of Fig. 2a). For critical phenomena, $\xi$ is scaled as $\xi \sim \left| {n_{2{\mathrm{D}}} - n_{\mathrm{c}}} \right|^{ - \nu }$, where $\nu$ is the correlation length exponent. To extend the Efros–Shklovskii theory to the region near MIT, a phenomenological scaling ansatz for $\varepsilon$ is adopted as $\varepsilon \sim \xi ^{\eta - 1}\sim \left| {n_{2{\mathrm{D}}} - n_{\mathrm{c}}} \right|^{ - \zeta }$, implying that Coulomb interactions become weaker near the transition region when approaching from the insulating phase^19, 20^. Thus, $T_{{\mathrm{ES}}}\sim \left| {n_{2{\mathrm{D}}} - n_{\mathrm{c}}} \right|^{\eta \nu }$, where $\eta$ is defined by $\zeta \equiv \nu \left( {\eta - 1} \right)$ and usually 1<*η*<3. Figure 2b shows the power law behavior for *T*_ES_ yielding $\eta \nu =$2.60, supporting the criticality.

In low-temperature and high-field regime, $E = V_{{\mathrm{ch}}}/L \gg k_{\mathrm{B}}T/q\xi$ (*L* is the channel length), the conduction is driven by a field with a negligible thermal effect. In the presence of Coulomb gap, the field-driven conductivity in insulating phase is described as^21, 22^

2 $$\sigma \left( E \right) \propto {\mathrm{exp}}\left[ {\left( { - \frac{{E_{{\mathrm{ES}}}}}{E}} \right)^{1{\mathrm{/}}2}} \right],$$

where $E_{{\mathrm{ES}}} = \frac{{k_BT_{{\mathrm{ES}}}}}{{2q\xi }}$. Here *T*_ES_ is the same parameter as in Eq. (1). Figure 2c shows *E*-dependent conductivity, which is fitted by Eq. (2). The higher field regions deviate from fitting lines due to the group velocity saturation. The fitting parameter *E*_ES_ is displayed in the inset of Fig. 2c. In principle, the localization length $\xi$ can be determined from two fitting parameters *T*_ES_ and *E*_ES_ using Eq. (2). However, as it approaches to the transition, Eq. (2) applies rather poorly due to possibly partial heating effects and inapplicability of Eq. (2)^23^ near MIT, which prevents from determining the accurate $\xi$. Figure 2d shows the results in log–log scale. Since data points are scattered, it is not evident to determine $\nu$. For the guideline, we draw two lines as in Fig. 2d, which give $\nu$ $\approx$2.34 (red color) and 1.80 (blue color). In this $\nu$ range, $\eta$ = 1.11 ~ 1.44, and consequently $\zeta =$ 0.26 ~ 0.80. From $\varepsilon \sim \left| {n_{2{\mathrm{D}}} - n_{\mathrm{c}}} \right|^{ - \zeta }$, smaller $\zeta$ indicates that the dielectric screening in insulating phase is lower near the phase transition. As we will discuss later, this lower screening in insulating phase would be one of the reasons for the asymmetry in critical exponents around MIT.

### Scaling analysis

**Fig. 3:**
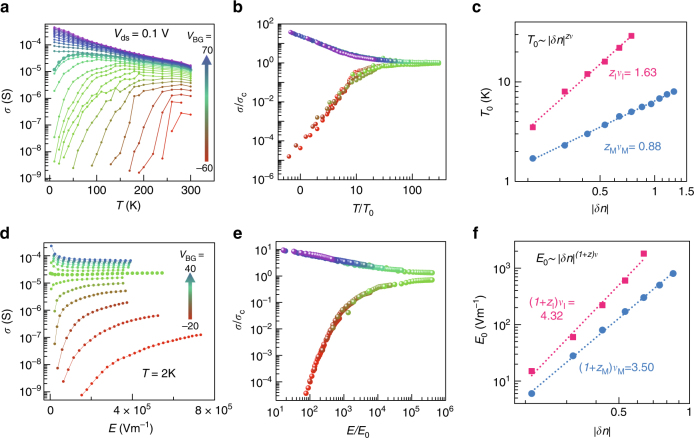
Scaling analysis for *T* and *E*. **a** $\sigma$ vs. *T* for several *V*_BG_*’*s at *V*_ds_ = 0.1 V **b** Renormalized conductivity $\sigma /\sigma _{\mathrm{c}}$ by the conductivity at $n_{\mathrm{c}}$ as a function of rescaled temperature *T/T*_0_. **c** Temperature scaling parameter *T*_0_ vs. $\left| {\delta n} \right|$, where $\delta n = \left( {n_{2{\mathrm{D}}} - n_{\mathrm{c}}} \right)/n_{\mathrm{c}}$. **d** $\sigma$ vs. *E* for several *V*_BG_*’*s at 2 K. **e** Renormalized conductivity $\sigma /\sigma _{\mathrm{c}}$ by the conductivity at $n_{\mathrm{c}}$ as a function of rescaled electric field *E/E*_0_. **f** Electric field scaling parameter *E*_0_ vs. $\left| {\delta n} \right|$

Scaling behavior is one of the hallmarks of a second-order phase transition in which the correlation length diverges at the transition. In quantum case, scaling the system size is equivalent to scaling the temperature. Figure 3a shows the temperature-dependent conductivity for several *V*_BG_ at a fixed *V*_ds_ = 0.1 V. The conductivity at *V*_BG_ = 10 V is chosen as the critical conductivity $\sigma _{\mathrm{c}}$ for renormalization (closed circles). At finite temperatures, the effective sample size $L_{{\mathrm{eff}}}\sim T^{ - 1/z}$, leading to the scaling for conductivity ${\mathrm{\sigma }}\left( {T,\delta n} \right)/\sigma _{\mathrm{c}}(T) = F_{\mathrm{T}}\left[ {T/T_0(\delta n)} \right]$, where *F* is universal scaling function, ${\mathrm{\delta }}n \equiv \left( {n_{2{\mathrm{D}}}/n_{\mathrm{c}} - 1} \right)$, $T_0\left( {\delta n} \right)\sim \left| {\delta n} \right|^{z\nu }$ and *z* is dynamical exponent. This equation implies that the renormalized conductivity $\sigma /\sigma _{\mathrm{c}}$ for different $n_{2{\mathrm{D}}}$ (or *V*_BG_) near the transition collapses into a single curve after the proper rescaling of temperature for each $n_{2{\mathrm{D}}}$, and the scaling parameter $T_0$ should follow the power law^24^ for $\delta n$ with the exponent $z\nu$. This scaling yields two collapsed branches for metallic (upper) and insulating phases (lower) (Fig. 3b). $T_0$ is given in log–log scale and the exponents $z\nu$ is determined by linear fits (Fig. 3c).

One remarkable feature is the asymmetrical values of $z{\mathrm{\nu}}$ for metallic ($\approx$ 0.88) and insulating phase ($\approx$ 1.63). This difference is too large to be erroneous. A similar small value $z\nu =$ 0.6 was reported for metallic phase in multilayer ReS_2_^25^. In general, critical exponents are independent of microscopic details but mainly subject to the symmetry of Hamiltonian and the dimensionality due to the diverging characteristic length scale near MIT. This universality is known to also be influenced by disorder and the range of Coulomb interactions. Experimentally, there seems to be a tendency that $z{\mathrm{\nu }}$ increases with disorder^11, 26^. The screening or Coulomb interactions can be also modulated across MIT, which can lead to asymmetric scaling behavior^27, 28^. Further discussion will be presented in a later section.

Besides this temperature scaling (*T*-scaling), the nonlinear transport property near MIT for an electric field *E* allows for electric field scaling (*E*-scaling)^29^ with an associated characteristic length $\ell _{\mathrm{E}}\sim E^{ - 1/(1 + z)}$, which strongly corroborates the quantum phase transition. This leads to the scaling form, $\sigma \left( {E,\delta n} \right)/\sigma _{\mathrm{c}}(E) = F_{\mathrm{E}}\left[ {E/E_0(\delta n)} \right]$, where $E_0\left( {\delta n} \right)\sim \left| {\delta n} \right|^{(1 + z)\nu }$. Figure 3d reproduces the electric field-dependent conductivity, which yields *E*-scaling (Fig. 3e). The scaling parameter $E_0$ for $\delta n$ is plotted in Fig. 3f. The extracted exponent, $\left( {1 + z} \right)\nu$ = 3.50 for metallic and 4.32 for insulating regime. From these two scaling analyses, exponents $\nu$ and $z$ are separately determined; $\nu _{\mathrm{M}}$ = 2.62 and $z_{\mathrm{M}}$ = 0.34 for metallic regime, while $\nu _{\mathrm{I}}$ = 2.69 and $z_{\mathrm{I}}$ = 0.61 for insulating regime.

### Hot electron effect and the relevance of *E*-scaling

At this point, we point out that *E*-scaling should be taken into account with caution. The critical exponents in *E*-scaling may deviate if there is a significant heating effect on electrons^30^. In general, the input power *P* = *IV* is used to make electrons hot if no phonons are generated. Energy of hot electrons is dissipated by releasing heat to phonons. At low temperature, electron–phonon coupling is reduced by the reduced phonons and thus hot electrons can be generated more easily than high temperature. This effect is expressed by a following power law relation,

3 $$P = IV = g\left( {T_{\mathrm{e}}^\alpha - T^\alpha } \right),$$

where $g$ is the effective coupling constant and $\alpha$ exponent of the power law. In disordered thin film including monolayer MoS_2_ at low temperature, where only acoustic phonons are relevant, i.e., below Bloch Grüneisen temperature, it has been observed that $\alpha = 4\sim 6$^31–34^. This value decreases with temperature increasing. For example, this value becomes 1 in monolayer MoS_2_^33^.

**Fig. 4:**
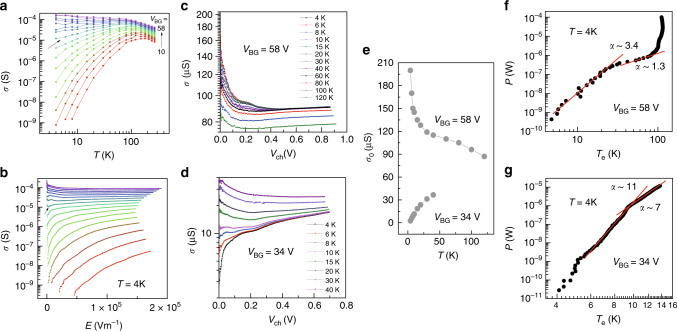
Field-induced hot electron effect. **a** $\sigma$ vs. *T* for several *V*_BG_*’*s at *V*_ds_ = 0.2 V. **b** $\sigma$ vs. *E* for several *V*_BG_*’*s at 4 K. **c**, **d** $\sigma$ vs. *V*_ch_ for several temperatures at *V*_BG_ = 58 V and *V*_BG_ = 34 V, respectively. **e** Conductivity $\sigma _0$ in *V*_ch_ $\to$ 0 limit for each temperature from (**c**) and (**d**). **f**, **g** Power *P* = *IV* to make electron hot with respect to the effective electron temperature *T*_e_ at *V*_BG_ = 58 V and *V*_BG_ = 34 V, respectively. Arrows in (**a**) and (**b**) indicate $\sigma$ at critical backgate bias *V*_c_ = 37 V

In order to check the relevance of *E*-scaling in obtaining the critical exponents in multilayer MoS_2_ systems, we fabricated another MoS_2_ device of 3.5 nm thickness and performed *E*-scaling at wide temperature range (4K-300K) with many more data points (See Supplementary Note 3). Figures 4a, b show the temperature-dependent conductivity for several backgate biases and electric field-dependent conductivity at *T* = 4 K, respectively. The arrows in these figures indicate the traces corresponding to the critical field *V*_c_ = 37 V ($n_{\mathrm{c}}\sim 2$.65 × 10^12^ cm^−2^). From these two measurements, we again determine the critical exponents $z_{\mathrm{M}}\nu _{\mathrm{M}} =$ 1.33, $z_{\mathrm{M}} =$ 0.56, and $\nu _{\mathrm{M}} =$ 2.37 for metallic phase and $z_{\mathrm{I}}\nu _{\mathrm{I}} =$ 2.06, $z_{\mathrm{I}} =$ 0.80, and $\nu _{\mathrm{I}} =$ 2.58 for insulating phase. We obtain the qualitatively similar features to the first MoS_2_ sample, asymmetric $z\nu$ values and small $z$ (<1) value. Also, we find that this system is well described by Efros and Shklovskii variable range hopping (*ES*-hopping) (See Supplementary Note 3). To demonstrate the hot electron effect, we choose two electric field-dependent conductivity traces, one in metallic corresponding to *V*_BG_ = 58 V and the other in insulating phase corresponding to *V*_BG_ = 34 V. Figures 4c, d show the conductivity behaviors of these two traces as a function of *V*_ch_ at several different temperatures. The zero voltage limit of conductivity $\sigma _0$ for each temperature is shown in Fig. 4e. Based on the observed $\sigma _0$, we convert the voltage (or *E* field)- dependent conductivity at *T* = 4 K to the effective electron temperature *T*_e_. Figures 4f, g show the injected power as a function of *T*_e_ for *V*_BG_ = 58 V and *V*_BG_ = 34 V, respectively.

For metallic phase (Fig. 4f), the exponent $\alpha \sim 3.4$, which is close to 4 at low temperature (<*T*_BG_ ~ 20 K^35^, Bloch Grüneisen temperature). At high temperature (>20K), $\alpha$ is 1.3, close to 1, which is predicted in monolayer MoS_2_. Therefore, the possibility of contribution from hot electron effects cannot be excluded in metallic phase. The non-monotonic behavior at *T*_e_ $\ge$ 100 K in Fig. 4f implies additional effects and remain elusive at current stage. On the other hand, $\alpha$ ~ 11 in the insulating phase as shown in Fig. 4g is too large to have any significant hot electron effects.

There is one more requirement for *E*-scaling to be valid. *E*-scaling is based on the standard scaling theory of localization for disorder-driven transitions. In principle, this should hold in the diffusive regime, i.e., lowest temperature. Since 2 K or 4 K is just a small fraction of the Fermi temperature (150 ~ 200 K) and lower than Dingle temperature *T*_D_ ~ 10 K in our samples, the diffusive regime appears to be accomplished. On the other hand, *T*-scaling is generally accepted to hold for the interaction-driven transition also and unsubject to hot electron effects as long as the drain-source voltage *V*_ds_ (or channel voltage *V*_ch_) is small. We used *V*_ds_ = 0.1 V, which results in nearly zero channel voltage limit (See Supplementary Note 3). Hence, the fact of asymmetric critical exponents $z\nu$ across the MIT is affirmative regardless of *E*-scaling. However, the separation of $z$ and $\nu$ at least for the metallic phase is not reliable due to the heating effect. Even in insulating phase, each value of critical exponents should be taken with a caution. We will discuss this issue in the discussion section.

### Screening and asymmetric critical exponents

The change in screening properties across the MIT has been suggested for an asymmetric $\nu$^28, 36^. It is argued that $z =$2 for the non-interacting case or short-range interactions and $z =$1 for long-range interactions for superconductor-insulator transition^37–40^. Although this argument for the $z$ value may not be exact for the interacting 2D MIT case^41, 42^, it appears that both $z$ and $\nu$ are susceptible to the range of interaction.

The screening effect is generally stronger in metallic phase. Moreover, an apparent intermediate metallic feature with a power law-dependent conductivity, in which screening is expected to be poor, is not clearly visible or exists in a very narrow range^10^ (See Supplementary Note 4 for more details). This characteristic with poor screening in insulating phase even near MIT appears to result in an asymmetric scaling property. The possibility of a drastic change in critical phenomena across the MIT due to the change of screening was noted early by Mott^27^. However, it has not been discussed by most conventional theories for its importance. It requires further theoretical and experimental studies. In contrast, monolayer MoS_2_ shows significantly more symmetry, which is ascribed to the severer disorder effect. In other words, the MIT in monolayer MoS_2_ is more likely disorder-driven. However, the possibility of inaccuracy inherent from rather high drain-source voltage (0.5 V) especially at low temperature in scaling analysis for monolayer cannot be excluded (See Supplementary Note 5 for more details).

## Discussion

**Fig. 5:**
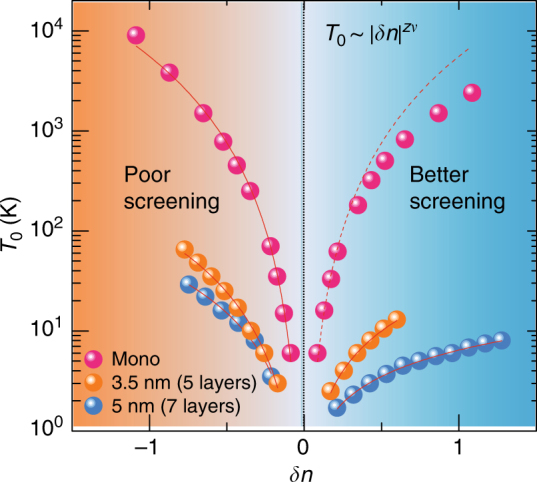
Asymmetric critical exponent. *T*_0_ vs. $\delta n$ for monolayer and multilayer MoS_2_. Red solid lines are power law fits to yield critical exponents $z\nu$ and dashed line is the symmetric counterparts to the insulating phase

The distinct features of two multilayer MoS_2_ with additional monolayer MoS_2_ are demonstrated in Fig. 5. $z\nu$ value increases and asymmetry is reduced as the thickness decreases. The origin is not clear but it is consistent with aforementioned experimental observations that $z\nu$ tend to increase in more disordered systems, or they are in a different universality class. The asymmetric critical exponent is interpreted as a result of different screening across the MIT, which naturally indicates the importance of correlation in this system. In addition, relatively small field effect mobility entails the soft Coulomb gap description, and data well fitted by *ES*-hopping transport in the insulating phase support this description. One important feature here is the power law behavior of *T*_ES_ as in Fig. 2b, which demonstrates the criticality of MIT as the evidence opposing to the viewpoint of crossover phenomena between strong and weak localization effects at rather high temperature for 2D MIT^43^.

**Fig. 6:**
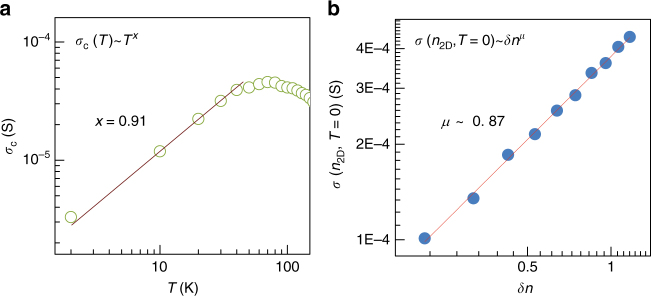
Conductivity at critical density and zero temperature for 5 nm thick MoS_2_. **a** Critical conductivity $\sigma _{\mathrm{c}}$ at critical carrier density as a function of temperature. **b** Conductivity in the limit of zero temperature as a function of $\delta n = \left| {n_{2{\mathrm{D}}} - n_{\mathrm{c}}} \right|/n_{\mathrm{c}}$ for metallic phase

There is an approach to check the self-consistency for the quantum phase transition. The critical conductivity $\sigma _{\mathrm{c}}$, in general, has the power law with temperature as $\sigma _{\mathrm{c}}\sim T^x$. From the Wegner scaling, it is expected that $x = \left( {{\mathrm{d}} - 2} \right)/z$. Thus, *x* = 0 for two dimension (*d* = 2), i.e., $\sigma _{\mathrm{c}}$ is temperature-independent. However, it was argued that $x \ne 0$ does not contradict any fundamental principle for 2D systems^44, 45^. Usually, when strong disorder is introduced into a system, it was experimentally shown that the relation $\mu = x(z\nu )$ is applied^11, 44^, where $\mu$ is the exponent in the relation $\sigma \left( {n_{2{\mathrm{D}}},T = 0} \right)\sim \delta n^\mu$. Here, $\sigma \left( {n_{2{\mathrm{D}}},T = 0} \right)$ is the conductivity in the limit of zero temperature. For a low disorder system, it has been experimentally known that *x* is usually indecisive and $\mu \approx$1–1.5^45, 46^. We have applied the relation $\mu = x(z\nu )$ to our 5 nm thick MoS_2_. We obtained *x* ~ 0.91 and $\mu$ ~ 0.87. The value $x\left( {z\nu } \right) =$0.91×0.88 = ~ 0.80 agrees well with $\mu$ ~ 0.87 as shown in Fig. 6.

Finally, we discuss the value of $z$. The dynamic critical exponent $z$ is defined by $\tau _c\sim \xi ^z$, physically describing how fast the system restores equilibrium against fluctuation, where $\tau _c$ is the characteristic time for the decay of fluctuation. As the transition approaches, $\tau _c$ increases with a diverging $\xi$, i.e., the system slows down. Thus, a smaller value of $z$ indicates faster equilibrium. However, *z* < 1 as in our multilayer MoS_2_ is unusual based on the majority theories that evaluate $z \ge 1$ for most phase transitions. For the 2D MIT case, it is shown that the compressibility $\chi$ in the presence of Coulomb gap vanishes at the transition as it approaches from metallic phase^47^. Since $\chi \sim \delta n^{\nu (2 - z)}$, *z* is bounded by *z* < 2 with an unknown lower bound. In a 2D superconductor-insulator transition, it is argued that the relevance of long-range Coulomb interaction requires *z* = 1^40^. The theoretical finding of *z* < 1 is attributed to the emergence of new low energy excitation^48^. At current stage, the origin of *z* < 1 in multilayer MoS_2_ is unidentified. Perhaps, *E*-scaling requires the lower temperature than 4 K to be valid in strong correlation or it is a new class of universality in this Mott–Anderson transition type. This requires further study. Assuming that our *E*-scaling is unreliable due to some non-negligible heating effects or the measurement at rather high temperature to be in diffusive regime, and thus, in fact, *z* ≳ 1, we can exclude a disorder-dominated transition in this thick multilayer MoS_2_ since $z_{\mathrm{M}}\nu _{\mathrm{M}} =$ 0.88, which is congruent with previous report of small value (0.6) in a few layer ReS_2_^25^, violates Harris criterion^49, 50^, i.e., $\nu _{\mathrm{M}}$ < 1. For thinner multilayer MoS_2_, $z \approx$ 0.80 (for insulating phase) seems quite close to 1 considering all the difficulties in *E*-scaling.

When both disorder and interaction effects are important, it was predicted that the metallic glass phase can appear with the power law temperature dependence^9^, i.e., $\sigma _{\mathrm{c}}\left( {n_{\mathrm{c}},T} \right) \propto T^{3/2}$, and experimentally observed^10, 44^. In our multilayer MoS_2_, such phase seems to possibly exist in a narrow window of a carrier density, which suggests an interaction-dominated transition also. This Mott-like transition in multilayer MoS_2_, which is quite strongly disordered in terms of the field effect mobility, supports the theoretical prediction that disorder tends to be screened by correlation effects^13^.

In summary, we observed MIT in multilayer MoS_2_. In this system, disorder and interactions are both important, leading to soft Coulomb gap picture to describe hopping transport in insulating phase. However, the metallic glass feature is rarely seen possibly due to strong interaction effects. The *T*-scaling analysis and a criticality in localization length are consistent with quantum phase transition. The asymmetric critical behavior across the MIT is interpreted as a result of asymmetric screening. Although *E*-scaling is uncertain for its validity, the clear feature of MIT in electric field-dependent conductivity is in harmony with quantum phase transition. While the transition in multilayer MoS_2_ is likely interaction-dominated, a disorder-dominated transition is speculated in monolayer MoS_2_.

## Methods

### Device fabrication and electrical transport measurement

A multilayer MoS_2_ flake was mechanically exfoliated on a highly doped 300 nm SiO_2_/Si substrate. To pattern the electrodes, PMMA A4 was spin-coated (3000 rpm, 50 s) and then, the electron beam lithography (EBL) was performed, followed by Cr/Au (2/60 nm) evaporation in high vacuum (~10^−6^ Torr). For monolayer MoS_2_ device, multilayer (15–20 nm thick) h-BN flakes were, first, mechanically exfoliated on a highly doped 300 nm SiO_2_/Si substrate, and then a CVD-grown monolayer MoS_2_ flake was transferred onto it. The post processes, EBL and metal evaporation, were the same as those of the multilayer MoS_2_ device. The dimensions of multilayer MoS_2_ (thickness = 5 ${\mathrm{n}}$m, length = 7.1 ${\mathrm{\mu }}$m, and width = 6 ${\mathrm{\mu }}$m for first sample and thickness = 3.5 nm, length = 11.1 ${{\mu }}$m, and width = 6.5 ${\mathrm{\mu }}$m for second sample) and monolayer MoS_2_ device (length = 12 ${\mathrm{\mu }}$m and width = 10.4 ${\mathrm{\mu }}$m) were confirmed by an atomic force microscope (SPA 400, SEIKO).

Four-probe electrical measurements were performed in high vacuum (~10^−^^6^ Torr) using a commercial semiconductor characterization system (4200-SCS, Keithley) for monolayer MoS_2_, and a cryostat (PPMS, Quantum Design, Inc.) with a characterization system (B1500A, Keysight Technologies).

### Data availability

The data supporting this study are available from the corresponding authors upon request.

## Electronic supplementary material

Supplementary Information
