# Supplementary material for: Soft Coulomb gap and asymmetric scaling towards metal-insulator quantum criticality in multilayer MoS2
Source: Nat Commun. 2018 May 24;9:2052. doi: 10.1038/s41467-018-04474-4 (PMC5967350; doi:10.1038/s41467-018-04474-4)
Supplement: Supplementary file 1 — Supplementary Information [file 41467_2018_4474_MOESM1_ESM.pdf]

Observing metal-insulator quantum criticality in multilayer MoS_2: Soft Coulomb gap and asymmetric scaling

Moon et al.

Supplementary Information

Observing metal-insulator quantum criticality in multilayer MoS_2: Soft Coulomb gap and asymmetric scaling

Byoung Hee Moon[1,2†]*, Jung Jun Bae [1†], Min-Kyu Joo[1], Homin Choi[1,2], Gang Hee Han[1], Hanjo Lim[3], Young Hee Lee[1,2]*

[1]Center for Integrated Nanostructure Physics, Institute for Basic Science (IBS), Suwon 16419, Republic of Korea

[2]Department of Energy Science, Sungkyunkwan University, Suwon 16419, Republic of Korea.

[3]Institute for Basic Science (IBS), Daejeon 34047, Republic of Korea

Supplementary Note 1. Determination of carrier density

In order to check how reliable the "simple approximation" is, we made another multilayer MoS_2 device of similar thickness, ~ 4 nm as shown in the inset of Supplementary Fig. 1 below. Supplementary Fig. 1a shows the current response to the backgate bias V_{BG}. The threshold voltage V_{th} was determined as usual by extrapolating the linear part as in this figure, giving V_{th} ~11 V. For comparison, we performed Hall measurements. At each fixed magnetic field B from 0 to 8 T by 0.5 T step, we did backgate bias sweeps and measured Hall voltages. From the slope in V_{Hall} vs. B plot for each V_{BG}, we show an example for chosen backgate bias in Supplementary Fig. 1b and the carrier density n_{2D} for each V_{BG} was calculated as shown in Supplementary Fig. 1c. n_{2D} values are rather scattered but their V_{BG} dependences show the decent linear behavior for both V_{ds} = 0.5 and 1 V. The solid line in this figure is from the approximation $n_{2D} = C_{ox}(V_{BG} - V_{th})/q$ using V_{th} = 11 V and C_{ox} = 11.5×10^{-8} F/cm^2 for 300 nm SiO_2. The effective oxide capacitance from the Hall measurement is nearly identical to the geometric value while the carrier density obtained from geometry undereatimates the value of n_{2D}. However, we note that the determination of the critical exponent values is insensitive to the constant offset of

n_{2D} due to $\delta n \equiv (n_{2D} - n_c)/n_c$.

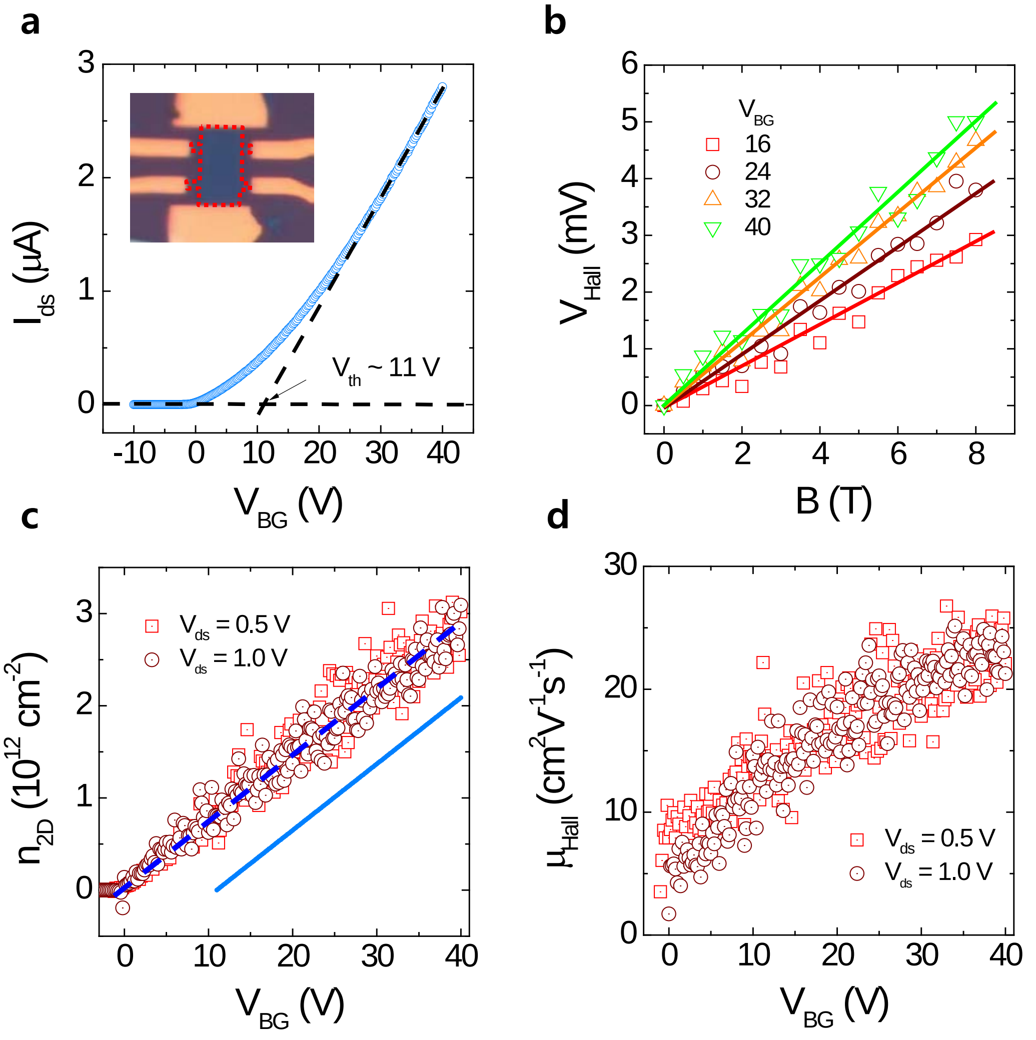

Supplementary Figure 1. (a) I_{ds} vs. V_{BG}. Inset: optical image of device. (b) Hall voltage V_{Hall} as a function of B for chosen backgate bias V_{BG}'s measured at V_{ds} = 1 V. (c) Carrier densities n_{2D} calculated from the slopes of (b) as a function of V_{BG} for V_{ds} = 0.5 and 1 V. (d) Hall mobility for V_{BG} at V_{ds} = 0.5 and 1 V.

Supplementary Note 2. 2- and 4-probe mobilities

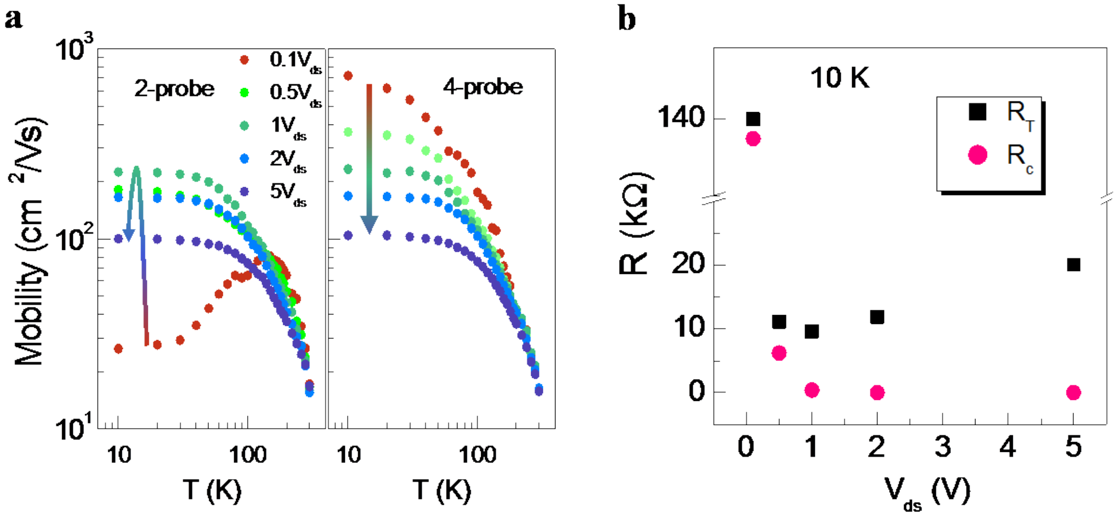

Supplementary Figure 2. (a) Temperature dependent 2-probe (left) and 4-probe (right) mobility for several drain-source voltage V_{ds}. (b) Voltage dependent total (R_{T}) and contact (R_{c}) resistance at 10K. Resistances were evaluated at backgate bias at which mobilities were calculated.

Supplementary Fig. 2a presents 2- (left) and 4-probe (right) field effect mobilities as a function of T for several V_{ds}'s, denoted as $\mu_{2\mathrm{FE}}$ and $\mu_{4\mathrm{FE}}$, respectively. Here, $\mu_{4\mathrm{FE}}$ is replotted for comparsion. $\mu_{2\mathrm{FE}}$were calculated from the maximal transconductance ($g_{\mathrm{m}} \equiv dI_{\mathrm{D}}/dV_{\mathrm{BG}}$) within the range of experimental V_{BG}. In general, $\mu_{2\mathrm{FE}} < \mu_{4\mathrm{FE}}$ due to the contact resistance R_{c}. The magnitudes of $\mu_{2\mathrm{FE}}$ and $\mu_{4\mathrm{FE}}$ for V_{ds} show quite different tendency. Focusing on the behavior at low temperature, $\mu_{2\mathrm{FE}}$ increases with V_{ds} up to 1V, and turns around to decrease as V_{ds} further increases. On the other hand, $\mu_{4\mathrm{FE}}$ keeps decreasing as V_{ds} increases. These contrasting behaviors originate from R_{c} and the dependence of σ on V_{ds}. First of all, Supplementary Fig. 2b shows the total resistance R_{T} (squares) and the contact resistance R_{c} (circles) at T = 10K, where $R_{\mathrm{T}} = R_{\mathrm{c}}+R_{\mathrm{ch}}$, R_{ch} is the channel resistance. At low V_{ds}, R_{c} is dominant due to the Schottky barrier. As V_{ds} increases, R_{c} becomes smaller and negligible above 1 V because the Schottky barrier becomes thinner. Consequently, $\mu_{2\mathrm{FE}}$ is determined mainly by R_{c} for low V_{ds} and R_{ch} for high V_{ds}. Since the maximal transconductance is found in the metallic phase, and σ for mobility

calculation decreases as V_{ds} increases in this metallic phase as shown in Fig. 1b of the manuscript, μ_{2FE} shows the non-monotonic behavior for V_{ds}. In contrast, since μ_{4FE} involves only the channel, it monotonically decreases as V_{ds} increases. In Supplementary Fig. 1a, the largest μ_{4FE} at 10K is ~720 $cm^2V^{-1}s^{-1}$ at V_{ds} = 0.1 V. This voltage itself is certainly not small to give the zero voltage limit of conductivity. However, according to Supplementary Fig. 2b, most voltage drop for 0.1 V occurs in the contact, and only ~2% in the channel, which is ~2 mV. Therefore, we believe that the zero voltage limit of μ_{4FE} is not far from ~720 $cm^2V^{-1}s^{-1}$, and also the temperature scaling analysis of conductivity obtained at 0.1 V is reasonable.

Supplementary Note 3. Scaling analysis for 3.5 nm-thick MoS_2

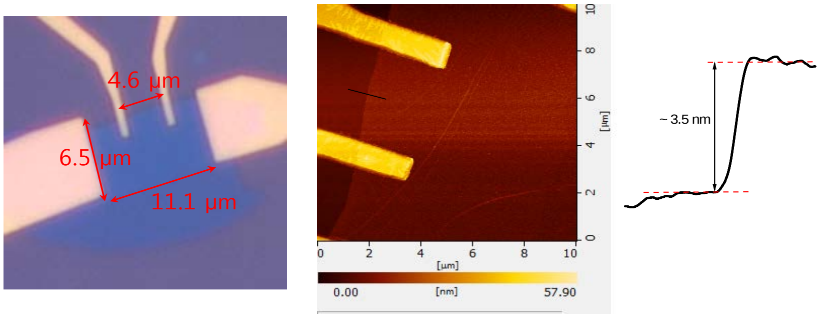

Supplementary Figure 3. Optical image of device (left), AFM image (middle), and thickness profile along the black line in AFM image (right).

We show the properties of 3.5 nm thick MoS_2 sample in this section. Supplementary Fig. 3 shows the optical and AFM images, and thickness profile of this sample.

Supplementary Fig. 4 shows conductivities for temperature (4a) and electric field (4d), and scaling analysis. The temperature 4K, where E-scaling is performed, is low enough to be in the diffusive regime, i.e., it is lower than the Dingle temperature $T_D = \hbar e/(2k_B m^* \mu) \sim$10K for $\mu \sim$1000 $cm^2V^{-1}s^{-1}$.

As shown in Supplementary Fig. 5, the conduction in the insulating phase is very well described by Efros-Shklovskii variable range hopping model down to the lowest temperature, 4K.

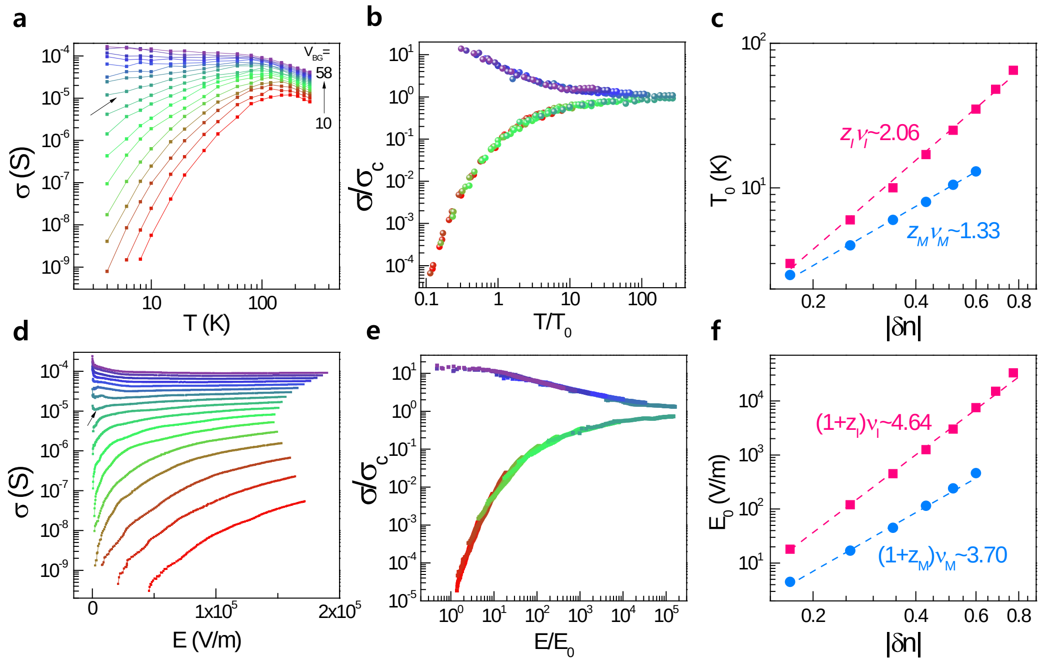

Supplementary Figure 4. **a** σ *vs.* T for several V_{BG}'s at V_{ds} = 0.2 V. **b** Renormalized conductivity $\sigma/\sigma_{\mathrm{c}}$ by the conductivity at n_{c} as a function of rescaled temperature T/T_0. **c** Temperature scaling parameter T_0 vs. $|\delta n|$. **d** σ *vs.* E for several V_{BG}'s at 4K. **e** Renormalized conductivity $\sigma/\sigma_{\mathrm{c}}$ by the conductivity at n_{c} as a function of rescaled electric field E/E_0. **f** Electric field scaling parameter E_0 *vs.* $|\delta n|$. Arrows in (a) and (d) indicate the traces at the critical field V_{c} = 37 V.

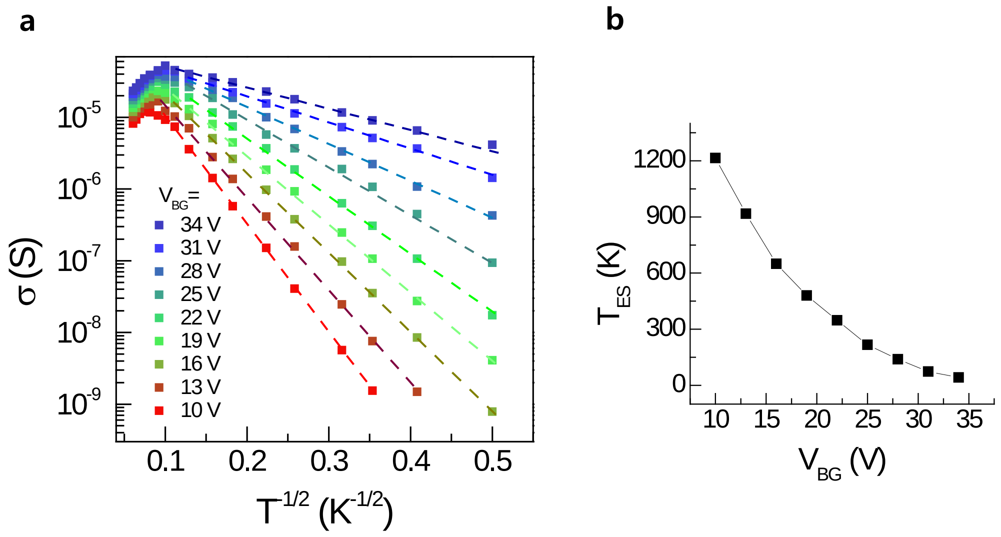

Supplementary Figure 5. **a** σ vs.$T^{-1/2}$ for Efros-Shklovskii hopping conduction. **b** Fitting parameters T_{ES}.

In the following, we show the simulation result for Joule heating of this device. It is performed using a COMSOL multiphysics modeling software. For the simulation, we used following parameters:

- sample thickness: 3.5 nm, Au electrode thickness: 60 nm.
- applied voltage to sample: 2 V
- sample thermal conductivity[1]: 60 W/m·K.
- thermal boundary conductivity between MoS_2 and SiO_2[2]: 14 MW/m^{2}K.

We simulate for three regions at 4K, i.e., insulating, near transition, and metallic phase. For simplicity, they are distinguished by the current level. Since the current level at transition point is ~ 1×10^{-5} A, we choose 8×10^{-6} A for insulating and 7×10^{-5} A for metallic region. According to the results in Supplementary Fig. 6, Joule heating for all three regions in our measurement configuration is insignificant.

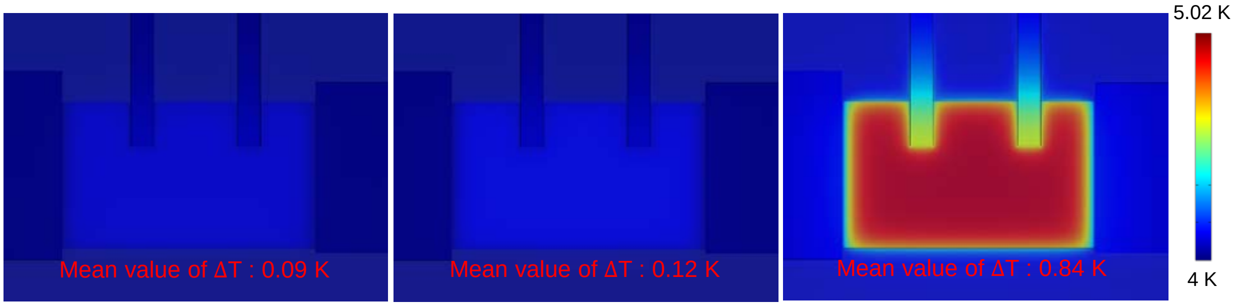

Supplementary Figure 6. Temperature rise (ΔT) due to Joule heating at T = 4K. For insulating region (left), $\Delta T \sim 0.09$K, near transition (middle), $\Delta T \sim 0.12$K, and metallic phase (right), $\Delta T \sim 0.84$K. These values are less than 20% independent of the regions.

To demonstrate that the excitation we used for the conductivity measurement in this device is close to the zero voltage limit so that the heating effect for temperature scaling is insignificant, we show two values in Supplementary Fig. 7 below, one in the zero voltage limit and the other at V_{ds} = 0.1 V. Supplementary Figs. 7a and 7d are the conductivities as a function of channel voltage V_{ch} for several temperatures at V_{BG} = 58 V for metallic and 34 V for insulating phase, respectively. Supplementary Figs. 7b and 7e are the expanded views of 7a and 7d near the zero voltage. The red dotted lines in these figures show the trend of conductivity change for extrapolating the conductivity in the zero voltage limit. Finally, we show two values in Supplementary Figs. 7c and 7f for V_{BG} = 58 V and 34 V, respectively. Closed circles are the values in the zero voltage limit, and open circles are the ones at V_{ds} = 0.1 V which were used for the temperature scaling. There are some underestimation for metallic phase and overestimation for insulating phase at the lowest temperature, but other than that, two values are quite similar. Since the temperature scaling was performed for the broad temperature range, we do not think around 10% error at the lowest temperature does cause the significant errors in the temperature scaling.

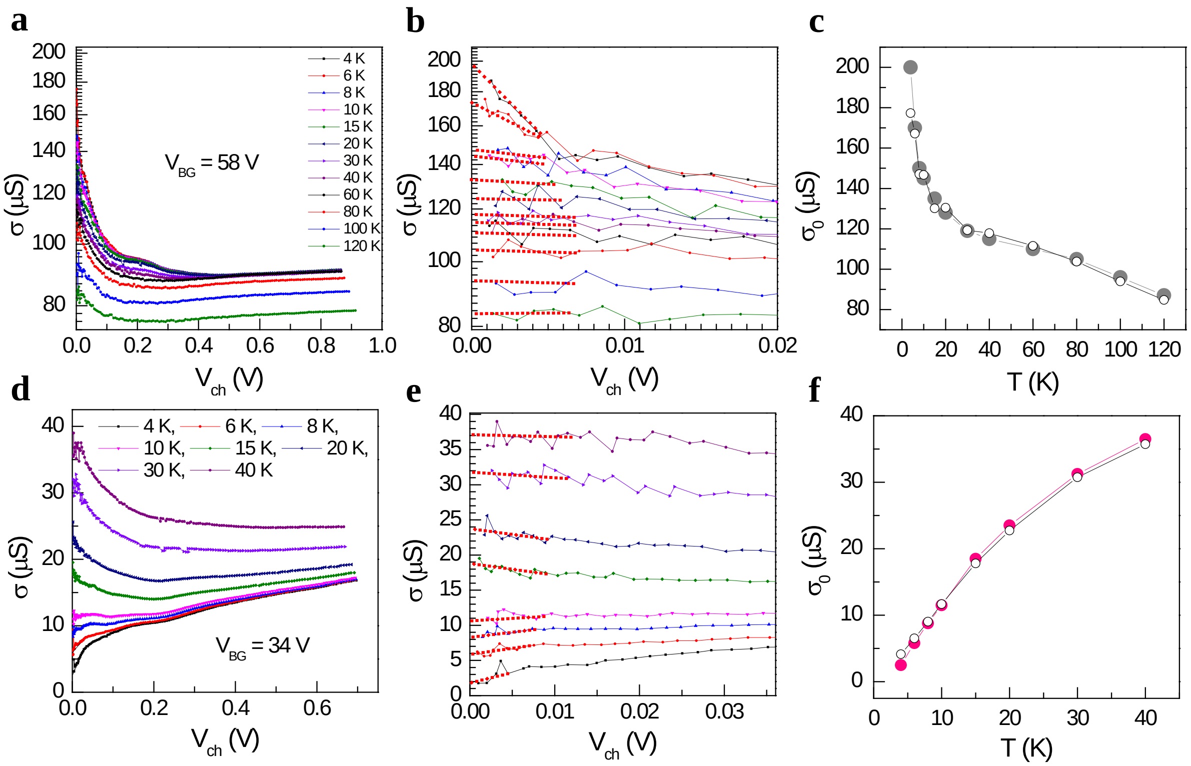

Supplementary Figure 7. **a** and **c** Voltage dependent conductivities at V_{BG} = 58 V and 34 V for several temperatures, respectively. **b** and **e** Expanded views of (a) and (d) near 0 V, respectively. Red dotted lines indicate converging trends to the conductivity in the zero voltage limit. **c** and **f** Solid circles are the conductivity values in the zero field limit and open circles are the values at V_{ds} = 0.1 V. V_{ch} corresponding to this V_{ds} value is $\lesssim$ 3 mV.

Supplementary Note 4. Intermediate glass phase

Electron glass features were experimentally observed in strongly disordered Si-MOSFET[3]. In the ref. S3, the critical carrier density $n_c \approx 5.2 \times 10^{11}$ cm^{-2}. $n_g \approx 7.5 \times 10^{11}$ cm^{-2} is identified as a carrier density below which the 2D electron system freezes into an electron glass. n_s^* is determined such that $d\sigma/dT = 0$ at this carrier density. The data close to n_c are well described by the power law behavior $\sigma(n_{2D}, T) = a(n_{2D}) + b(n_{2D})T^x$ with $x \approx 1.5$. This feature is consistent with the theoretical prediction of the existence of an intermediate glass phase in

$n_c < n_s < n_g < n_s^*$ (ref. 9 in the manuscript). In our data, we identified $V_{BG} = 10$ V as a critical field corresponding to $n_c \approx 3.37 \times 10^{12}$ cm^{-2}. The real critical field could be in between 10 and 15 V as shown in Supplementary Fig. 8 below. If it really is, the temperature dependence of σ would be weaker than the one at $V_{BG} = 10$ V . The power x for the trace at $V_{BG} = 10$ V is 0.91 far from 1.5, and the intermediate region (colored), if there is, very narrow, i.e., $\delta n_g \equiv |n_g - n_c|/n_c \approx 0.11$, which is contrast to the Si case in ref. S3, $\delta n_g \approx 0.44$. In this sense, an apparent metallic glass feature is not visible or exists in a very narrow range.

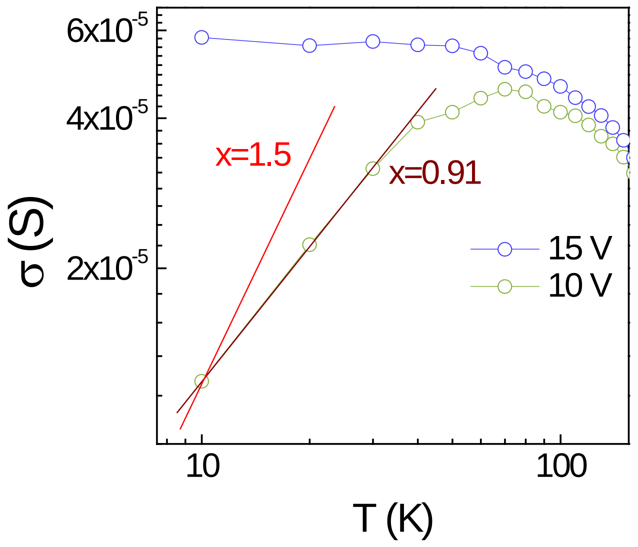

Supplementary Figure 8. σ vs. T for two traces at V_{BG}=10 and 15 V just near the MIT (5 nm thick MoS_2).

Supplementary Note 5. Scaling analysis for monolayer MoS_2

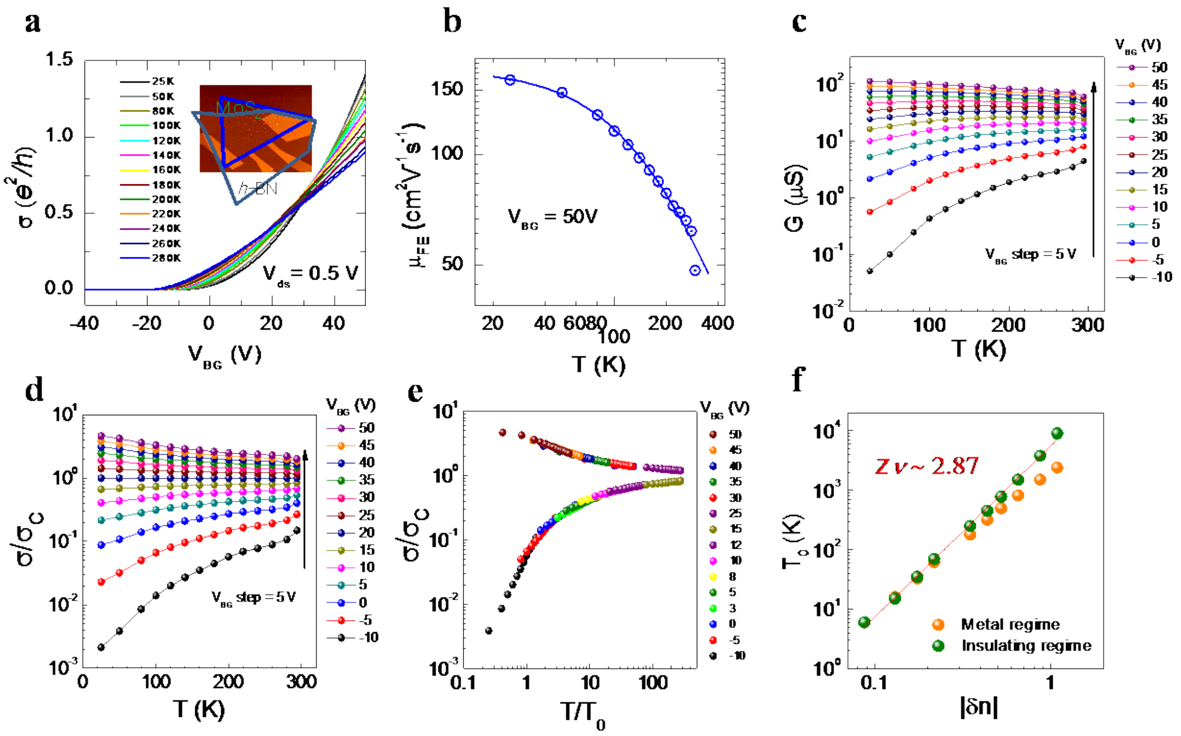

Supplementary Figure 9. **a** Backgate V_{BG} dependent conductivity in the unit of e^2/h at $V_{ds} = 0.5$ V for various temperatures. **b** Temperature dependent 4-probe mobility for $V_{BG} = 50$ V. **c** Conductance G as a function of temperature for various V_{BG}'s. **d** Renormalized conductivity σ/σ_c as a function of temperature for various V_{BG}'s. **e** Renormalized conductivity for rescaled temperature T/T_0. **f** Temperature scaling parameter T_0 vs. $|\delta n|$.

Temperature scaling was performed for monolayer MoS_2. CVD (chemical vapor deposition) grown monolayer MoS_2 is transferred onto the h-BN film (~20 nm) mechanically exfoliated on the SiO_2 (300 nm)/Si substrate. The optical image of device with metal (Cr/Au) electrodes is shown in the inset of Supplementary Fig. 9a. Supplementary Fig. 9a presents the backgate bias dependent conductivity in the unit of e^2/h for selected temperatures. This conductivity was taken at $V_{ds} = 0.5$ V which is not small but we believe that this does not change the scaling behavior significantly since our temperature range for scaling is rather high, T > 60K so that the field effect is relatively weak compared to the thermal effect. Conductivity crossover for temperature

occurs around V_{BG} ~ 23 V signifying the metal-insulator transition. The critical carrier density n_c at this bias is estimated to be ~1.9×10^{12} cm^{-2} using $n_{2D} = C_{ox}(V_{BG} - V_{th})/q$ at room temperature as in the manuscript. This n_c yields r_s~7.8.

Temperature dependent 4-probe mobility is calculated at V_{BG} = 50 V and shown in Supplementary Fig. 9b. The mobility at ~10K is approximately 170 $cm^2V^{-1}s^{-1}$. Compared with multilayer, monolayer MoS_2 is less interacting and more disordered system. Supplementary Figs. 9c and 9d display the conductance and renormalized conductivities by the critical conductivity as a function of temperature, respectively. Supplementary Fig. 9e shows the collapse of renormalized conductivities after rescaling the temperatures.

Finally, the critical exponent $z\nu$~2.87 is obtained from the linear fit of temperature scaling parameter T_0 for $|\delta n|$ as shown in Supplementary Fig. 9f. In addition to this large value, more symmetric scaling parameter T_0 for the metal and insulating phase suggest that MIT in this monolayer MoS_2 is likely disorder driven. The deviation at higher $|\delta n|$ in metallic regime is not clearly understood. The intermediate state may exist.

In another CVD-grown monolayer MoS_2 on h-BN, we measured voltage-dependent conductivity for several temperatures to see how much variation of conductivity for the voltage changes as the temperature increases. Supplementary Fig. 10a shows the optical image of monolayer MoS_2 on h-BN. Supplementary Fig. 10b displays the backgate bias-dependent conductivity for several different temperatures taken at V_{ds} = 0.5 V. The metal-insulator crossover is not visible for $V_{BG} \leq 80$ V but the larger curvature for smaller temperature indicates MIT to occur at higher V_{BG}. Supplementary Figs. 10c and 10d show drain-source voltage-dependent conductivity for different temperatures at V_{BG} = 35 V and 70 V, respectively. The conductivity changes with V_{ds}. The changing rate is stronger at lower temperature, while it becomes weaker as temperature increases. For T > 100K and V_{ds}<1 V, it seems that the voltage dependence of conductivity is rather weak. Since the scaling for monolayer was performed for T > 60K as noted earlier and the collapse (Supplementary Fig. 10e) is quite good up to 280K, this suggests that the scaling analysis with this voltage V_{ds} = 0.5 V is still reliable for insulating phase. Accordingly, for metallic phase, we expect the scaling analysis for monolayer to be still valid, although we could not explicitly proved it due to the inaccessibility of metallic phase in

additional experiments. Although the data for monolayer is not fully comprehensive, we believe it is worth reporting, since, to our knowledge, this is the first report for any kind of atomic monolayer form of materials.

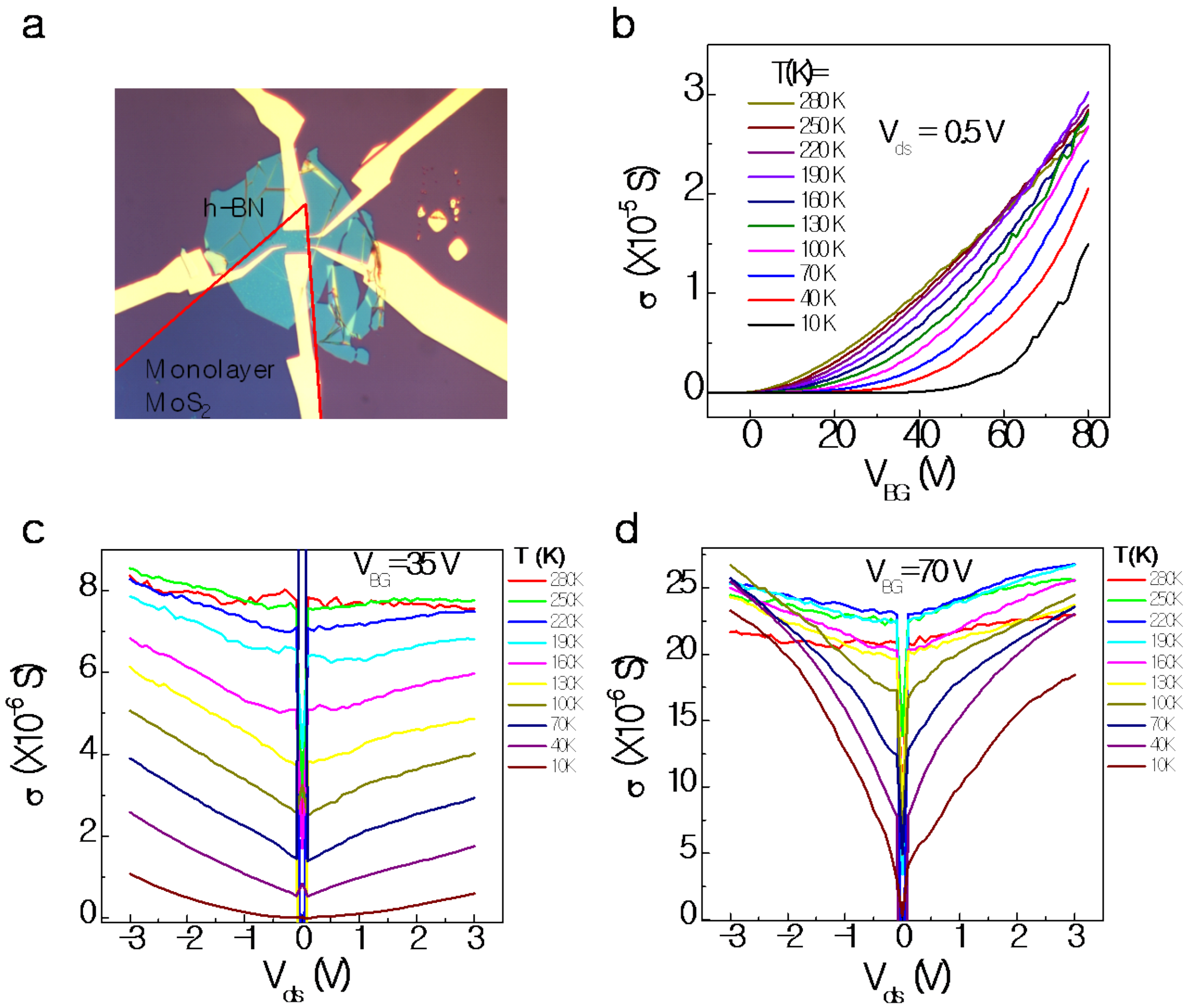

Supplementary Figure 10. a Optical image of monolayer MoS_2 on h-BN. **b** Backgate bias dependent conductivity for several temperatures taken at V_{ds} = 0.5 V. **c** Drain-source volate V_{ds} dependent conductivity for several temperatures at V_{BG} = 35 V and **d** 70 V.

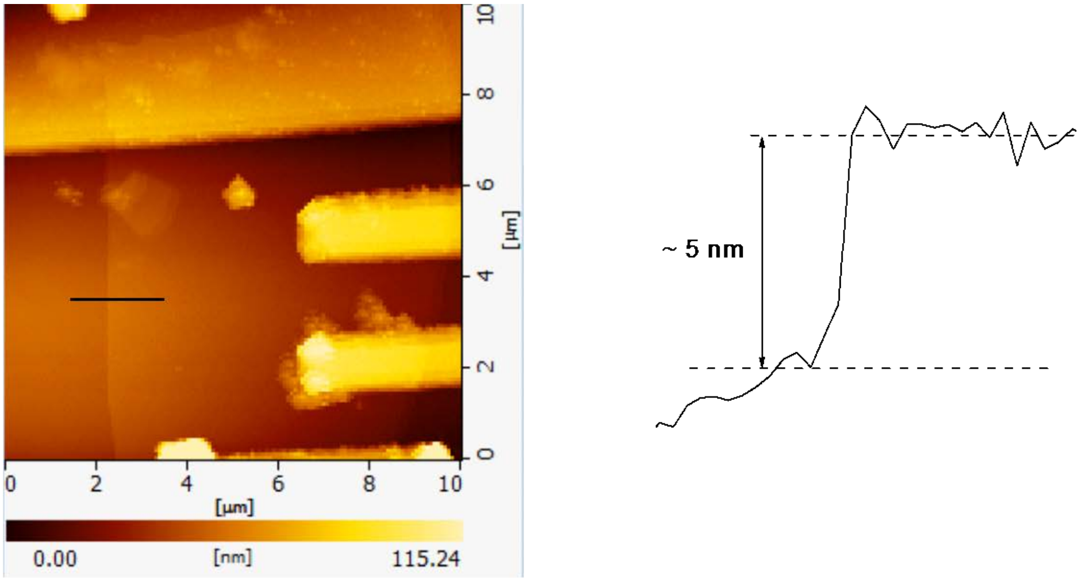

Supplementary Figure 11. AFM image of multilayer MoS_2 device (left) and thickness profile along the line.

Supplementary References

1. Bae J. J., *et al.* Thickness-dependent in-plane thermal conductivity of suspended MoS2 grown by chemical vapor deposition. *Nanoscale* **9**, 2541-2547 (2017).

2. Yalon E., *et al.* Energy Dissipation in Monolayer MoS2 Electronics. *Nano Lett.* **17**, 3429-3433 (2017).

3. Bogdanovich S., Popovic D. Onset of glassy dynamics in a two-dimensional electron system in silicon. *Phys. Rev. Lett.* **88**, 236401 (2002).
